# Prevalence and predictors of khat chewing among Ethiopian university students: A systematic review and meta-analysis

**DOI:** 10.1371/journal.pone.0195718

**Published:** 2018-04-12

**Authors:** Alemu Gebrie, Animut Alebel, Abriham Zegeye, Bekele Tesfaye

**Affiliations:** 1 Department of Biomedical Science, School of Medicine, Debre Markos University, Debre Markos, Ethiopia; 2 Department of Nursing, College of Health Sciences, Debre Markos University, Debre Markos, Ethiopia; Harvard Medical School, UNITED STATES

## Abstract

**Background:**

Khat chewing has become a common practice among university students in developing countries like Ethiopia. It has a potential effect on physical, mental, social and cognitive aspects of student functioning. In Ethiopia, study findings regarding the prevalence of khat chewing were highly dispersed and inconsistent. Therefore, this systematic review and meta-analysis estimates the pooled prevalence of khat chewing and its predictors among Ethiopian university students.

**Method:**

A systematic review and meta-analysis was conducted to assess the prevalence and predictors of khat chewing among university students in Ethiopia. We searched literature from the databases of PubMed, Google Scholar, Science Direct, and the Cochrane Library. A total of 24 Ethiopian studies reporting the prevalence of khat chewing among university students were included. Data were extracted using a standardized data extraction format prepared in Microsoft Excel and the analysis was done using STATA 14 statistical software. To assess heterogeneity, the Cochrane Q test statistics and *I*^*2*^ test were used. Since the included studies exhibit high heterogeneity, a random effect meta-analysis model was computed to estimate the pooled prevalence of khat chewing. Moreover, the association between predictor variables, and khat chewing practice were examined.

**Results:**

The meta-analysis of 24 studies revealed that the pooled prevalence of khat chewing among university students in Ethiopia was 23.22% (95% CI: 19.5, 27.0). In the subgroup analysis, the highest prevalence was observed in Oromia region (31.6%; 95CI: 21.2, 41.9) whereas the lowest prevalence was observed in Amhara region (18.1%; 95%CI: 12.4, 23.8). Being male OR: 2.76 (95% CI 1.64, 4.63), family khat chewing practice OR: 2.91 (95% CI 1.06, 7.98), friend khat chewing habit OR: 4.74 (95% CI 3.48, 13.06), alcohol drinking OR: 7.06 (95% CI 5.65, 8.82) and cigarette smoking habit OR: 15.11 (95% CI 8.96, 25.51) were found to be predictors of khat chewing.

**Conclusion:**

The study found that the prevalence of khat chewing among university students was quite common, with slightly more than 1 in 5 students engaging in the use of this substance. Being male, family khat chewing practice, friend’s khat chewing habit, alcohol drinking, and cigarette smoking were found to be predictors of khat chewing practice among university students.

## Introduction

Khat (Catha edulis) is an evergreen shrub that is planted and chewed in eastern through southern parts of Africa and the Arabian Peninsula [1]. It is believed that khat cultivation originated in Ethiopia, and was imported to Djibouti, Somalia, Kenya, Uganda, Tanzania, Zimbabwe, Zambia, South Africa and Yemen. In some of these countries, khat chewing goes far back in history and has always been a kind of tradition [2]. As reported from previous separate studies, it is evident that khat chewing by users is intended to increase concentration, self-confidence, creativity and imagination as well as communication abilities to associate ideas by virtue of its amphetamine-like activity with euphoric and stimulant effects [3, 4]. Khat chewing is becoming a common practice and great concern in university students because they think that it helps increase their academic performance especially during exams and for recreational purposes [5, 6].

The biochemically-active constituents of khat responsible for its psycho stimulant activity are the alkaloid chemicals called cathinone (first discovered in 1975 by the Laboratories of the United Nations) and cathine (a less potent form of cathinone) which are similar to the psychoactive substance amphetamine both structurally and functionally [7, 8]. Cathinone, a potent stimulator of the nervous system, causes activation of sympathetic as well as central nervous system activities similar to the pharmacological effects of amphetamine. Fresh leaves of the shrub contain both of the alkaloids, and the leaf if not refrigerated could lose its potency after 48 hours and would contain only cathine that explains the reason for the preference of users for fresh leaves of khat. Various experimental studies have indicated that khat as a substance could be recognized as a “natural amphetamine”. In low level, khat can result in loss of appetite, hyperactivity, euphoria, and enhanced intellectual efficiency [7, 9–11].

Despite the aforementioned effects of khat, its chronic and excessive chewing practice has been associated with serious social, psychological, sexual, economic, and health problems. High level and long-term consumption of khat have also resulted in various mental and neurological aberrations such as cognitive impairment, learning problems and behavioral abnormalities. In addition, different studies reported that besides its social and economic adverse effects, high levels of khat use have been associated with dental, cardiovascular, gastrointestinal and genitourinary problems [3, 12, 13]. Hence, WHO classified khat as a possible drug of abuse, though with less addictive potential than alcohol or tobacco [14].

Globally, it is estimated that 5–10 million people consume khat each day, although its use is largely confined to East African countries and south western Arabia [15, 16]. In Ethiopia, chewing of khat is becoming habitual and increasing at an alarming rate, especially in the younger segment of the population more importantly in high school, college and university students for whom academic activity is high. According to 2016 Ethiopia Demographic and Health Survey report, 12% of women and 27% of men reported having ever chewed chat [17].

In Ethiopian universities, different independent and fragmented studies have been conducted to assess the prevalence and predictors of khat chewing. These separate studies reported that the prevalence of khat chewing in Ethiopian university students ranged from 6.7% to56.8% [1, 6, 15, 18–42]. From the reports of these studies, there was a great variation and inconsistency related to the prevalence of khat chewing practice throughout the country [1, 6, 15, 18–41]. In addition to prevalence, socio-demographic (being male gender), and other predictors like peer pressure, family khat chewing practice, alcohol drinking, and cigarette smoking were the most common predictors reported by the Ethiopian studies [24, 25, 28, 32, 35].

The reasons for the above variation in the prevalence and predictors of khat chewing practice among Ethiopian university students have not yet been investigated. Therefore, the main aim of this systematic review and meta-analysis was to estimate the pooled prevalence of khat chewing practice and to identify its predictors among university students in Ethiopia. The findings of this meta-analysis will help policy makers and other concerned bodies to plan and campaign in combating the adverse consequences of habitual chewing of khat on health, and help educate the general population, especially the younger segment of the population. The study will also be of paramount importance for researchers to do in related topics. The review question is: What is the best available evidence on the prevalence and predictors of khat users in university students in Ethiopia?

## Methods

### Identification and study selection

Published and unpublished research reports on the prevalence and predictors of khat chewing among university students in Ethiopia were considered. Potentially relevant studies were identified through a literature search of MEDLINE/PubMed, Science Direct, EMBASE, HINARI and Cochrane Library. The searching of the articles was carried out from the 9^th^ of September, 2017 to the 5^th^ of November, 2017and all results were limited to English. Unpublished studies have been retrieved from the gray literature through Google and Google Scholar.

The key term used for the search were “Prevalence” OR “Epidemiology” AND “Khat chewing” OR “Chat chewing” OR “Substance abuse” AND “Ethiopian university”. All the literatures available until November5, 2017 were included in the systematic review and meta-analysis. The systematic review and meta-analysis was carried out in accordance with the Preferred Reporting Items for Systematic reviews and Meta-Analyses (PRISMA) guideline (**S1 Table**)[43].

### Eligibility criteria

We reviewed abstracts from initial search using defined inclusion and exclusion criteria.

### Inclusion criteria

**Study area:** Only articles conducted in Ethiopian universities.

**Study design**: All observational studies (cross-sectional, cohort and case controls) that contain original data reporting the prevalence and predictors of khat chewing among university students in Ethiopia were considered.

**Language**: Literatures published in the English language were included.

**Population**: Studies conducted among university students were considered

**Publication condition**: Both published and unpublished articles were considered

### Exclusion criteria

Before exclusion, we examined the eligibility of the studies by reading their titles and abstracts. After reading the abstracts, if the studies are relevant to our review, we examined the full texts. During the article selection process, studies, which were not fully accessed after reading the titles and abstracts, were excluded. However, before excluding the articles we attempted to contact the primary author at least two times through email. The reason for the exclusion of these articles is that we are unable to assess the quality of each article in the absence of their full texts. In addition, studies, which did not report our outcome of interest, were excluded after reviewing their full texts.

### Data abstraction

The two authors (AG and AA) independently extracted all the necessary data using a standardized data extraction format prepared in Microsoft Excel. For the primary outcome, the data extraction format included first author, region in the country where the studies were conducted, the university where the studies were carried out, publication year, study design, sample size, response rate and prevalence of khat chewing. For the second outcome (predictors), the data extraction format was prepared for each specific predictor (gender, family khat chewing habit, friend khat chewing practice, alcohol drinking habit, and cigarette smoking habit). We selected these variables because they are the most frequently reported predictors by the studies included in this met-analysis. In this study, we considered variables as a predictor (gender, family khat chewing habit, friend khat chewing practice, alcohol drinking habit, and cigarette smoking habit) if at least two or more studies reported them as a predictor. For each predictor, to calculate the odds ratio, we extracted the data from the primary studies in the form of two by two tables. Any disagreements between the two authors during extractions were discussed and solved through consensus.

### Outcome measurements

This systematic review and meta-analysis has two main outcomes. The primary outcome was to determine the prevalence of khat chewing among Ethiopian university students. The second outcome of the study was to identify the predictors of khat-chewing practice. The prevalence was calculated by dividing the number of participants engaged in khat chewing practice to the total number of participants who have been included in the study (sample size) multiplied by 100. Regarding predictor variables, we calculated the odds ratio from the primary studies using the two by two tables for more detail, see additional file (**S2 Table**).

### Quality assessment

To assess the quality of studies, we used the tool Newcastle-Ottawa Scale adapted for cross-sectional studies quality assessment [44]. The tool has indicators consisting of three main sections; the first section has five stars and assesses the methodological quality of each study. The second section of the tool evaluates the comparability of the studies. The last part of the tool measures the quality of the original articles with respect to their statistical analysis. Using the tool as a protocol, the two authors independently evaluated the qualities of the original articles. The quality of the studies was evaluated by using these indicators; those with medium (fulfilling 50% of quality assessment criteria) and high quality (≥6 out of 10 scales) were included for analysis. By taking the mean score of the two researchers, disagreements of their assessment results were resolved.

### Statistical analysis

The necessary data were extracted using a Microsoft Excel format and analyzed by using STATA Version 14.0 (software. The original articles were described using a table as well as forest plot. We calculated the standard error of prevalence for each original article by the binomial distribution formula. Heterogeneity among the reported prevalence of studies was checked by using heterogeneity χ2 test, I^2^test and the p-values [45].The above statistic tests indicated that there was a significant heterogeneity among the studies (I^2^ = 97.5%, p <0.001). Therefore, a random effects meta-analysis model was used to estimate the Der Simonian and Laird’s pooled effect. Moreover, univariate meta-regression model was conducted by taking the publication year and the sample size of the studies to identify the possible source of heterogeneity though none of them was statistically significant. Potential publication bias was also assessed objectively by using Egger’s correlation and Begg’s regression intercept tests at 5% significant level respectively [46, 47]. The results of these tests suggested that possible existence of a significant publication bias (p<0.001 in Egger’s test), the final effect size was determined by applying Duval and Tweedie's Trim and Fill analysis in the Random-effects model. In addition, to minimize the random variations between the point estimates of the primary study, subgroup analysis was done based on region of studies and sample size.

## Results

### Search results

**Fig 1:**
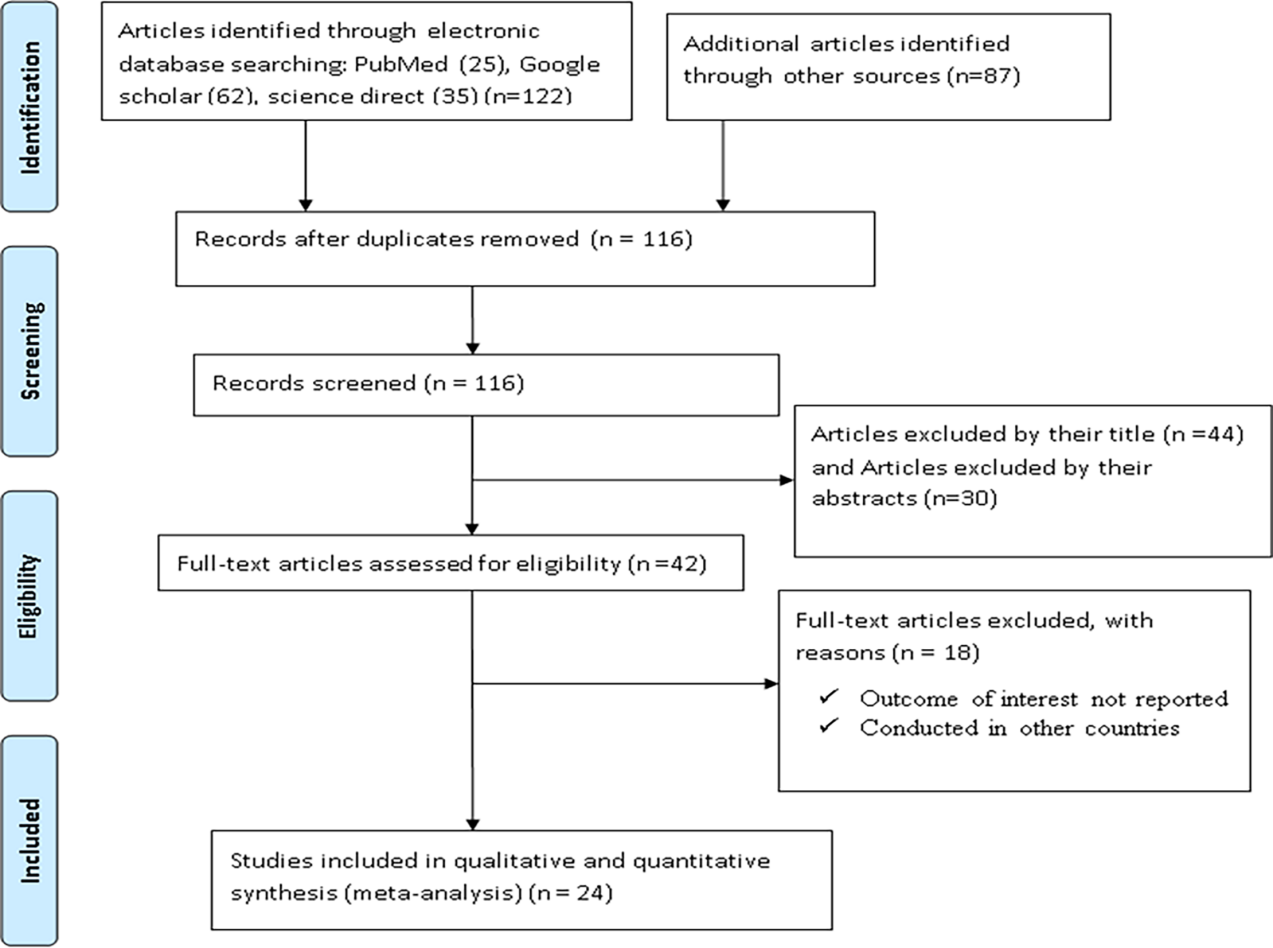
Flow chart describing the selection of studies for the systematic review and meta-analysis of prevalence and predictors of khat chewing among university students in Ethiopia, 2017 (identified screened, eligible and included studies). Articles may have been excluded for more than one reason.

In the first step of our search, 209 articles were retrieved regarding prevalence and predictors of khat chewing among university students using PubMed, Google scholar, science direct, HINARI, EMBASE, the Cochrane Library databases, and other sources described previously. Of these initial records, 93 articles were excluded due to duplication. From the remaining 116 articles, 74 articles were excluded after review of their titles and abstracts being assessed as non-relevant to this review. Therefore, 42 full text articles were accessed, and assessed for eligibility based on the pre-set criteria, which resulted in further exclusion of 18 articles primarily due to the study population and outcome of interest. Among these, six of the studies were conducted in Yemen [48, 49], Saudi Arabia[50], Sudan [51], Uganda[52], and Kenya [53]. The remaining twelve studies were conducted in different parts of Ethiopia [27, 35, 50, 54–62]and excluded because of the study population (conducted other than universities students) and unreported outcome of interest. Finally, 24 studies were purported to be eligible and included in the meta-analysis (**Fig 1**)

#### Characteristics of original articles

**Table 1**summarizes the characteristics of the 24 original articles included in this systematic review and meta-analysis. regarding study design, all of the articles are cross sectional studies, and the sample size ranged from 161 in Adigrat University [34] to 3872 in Bahir Dar University [24]. Moreover, these studies were conducted from 2009 and 2017. In the present meta-analysis, to estimate the pooled prevalence of khat chewing a total of 22,351 university students were involved. Concerning geographical distribution of the studies, the 24 studies were obtained from the seven regions of the country: one study from Addis Ababa [6], five from Oromia [18, 33, 37, 63, 64], seven from Amhara [19, 20, 24, 25, 28, 32, 36], three from Tigray [26, 34, 41], two from Harari [22, 39], one from Somali region [23], and five from Southern Nations, Nationalities and Peoples’ Region (SNNPR) [21, 29, 30, 38, 40]. However, there were no studies conducted in Benishagule Gumize, Afar, and Gambella regions of the country. The highest prevalence of khat chewing practice (56.8%) was reported in Ambo University (Oromia region) [64] whereas the lowest prevalence (6.67%) was reported from a study done in Adigrat University (Tigray region) [34]. In addition, the original studies included in the meta-analysis had a response rate ranging from 62.9% to 100% and almost all the studies had good response rate. Regarding the publication condition of the studies, three [41, 63, 64] of the 24 studies were unpublished, and 21 of the studies were published in reputable journals. Finally, the quality score of the studies ranges from 5–8 out of 10 points.

**Table 1:** Descriptive summary of 24 studies reporting the prevalence and predictors of khat chewing among university students in Ethiopia included in the systematic review and meta-analysis, 2017.

Region	University	Author	Publication year	Sample size	Response rate (%)	Quality score (10 pts)	Prevalence (95% CI)
Addis Ababa	Addis Ababa	Wakgari and Aklilu[6]	2011	800	78	6	14.00 (11.27,16.73)
Amhara	Gondar	Yalemzewod et al [19]	2017	800	92	7	16.44 (13.76,19.12)
	Bahir Dar	Ewenat et al [24]	2014	3872	77.5	6	23.99 (22.46,25.52)
	Gondar	Aklilu et al [28]	2014	310	97.4	6	9.60 (6.28,12.93)
	Gondar	Desalegn et al [32]	2014	682	62.9	5	24.48 (20.41,28.54)
	Woldia	Misgan et al [20]	2017	730	89.7	7	13.00 (10.42,15.58)
	Debre Markos	Girmay and Ahmed[36]	2014	845	96.6	7	28.50 (25.37,31.63)
	Bahir Dar	Ewenat et al [25]	2014	3872	84.4	6	11.00 (9.93,12.07)
Harari	Haramaya	Andualem et al [22]	2014	764	94.99	6	30.30 (26.95,33.65)
	Haramaya	Gezahegn et al [39]	2014	1040	98.3	7	23.60 (21.00,26.20)
Oromia	Jimma	Kanchi and Deribachew[37]	2015	634	97.8	5	17.74 (14.73,20.75)
	Jimma	Tilahun et al [18]	2017	651	95.1	8	23.91 (20.55,27.27)
	Jimma	Kalayu et al [33]	2009	248	92.7	7	33.00 (26.92,39.08)
	Adama	Getu T and Alemayehu M [63]	2012	764	95.3	7	27.70 (24.45,30.95)
	Ambo	Megersa G and Meseret K[42]	2014	264	100	7	56.80 (50.82,62.78)
SNNPR	Hawassa	Andargachew et al [30]	2016	590	94.5	7	16.31 (13.24,19.37)
	Hawassa	Ayalew et al [21]	2015	1290	97.3	7	10.52 (8.82,12.22)
	Hawassa	Andargachew et al [29]	2014	590	99.3	8	20.30 (17.04,23.56)
	WolaitaSodo	Tesfa et al [40]	2017	747	97.05	7	35.70 (24.45,30.95)
	Dilla	Moges[38]	2014	623	98.1	7	41.08 (37.18,44.98)
Somali	Jigjiga	Gamachu et al [23]	2017	648	92.6	6	33.33 (29.56,37.11)
Tigray	Mekele	Kidan[41]	2011	662	90.8	7	14.80 (11.96,17.64)
	Adigrat	Tilahun et al [34]	2015	161	93.17	5	6.67 (2.67,10.66)
	Axum	Measho et al [26]	2013	764	98.7	7	28.70 (25.48,31.93)

### Meta-analysis

**Fig 2:**
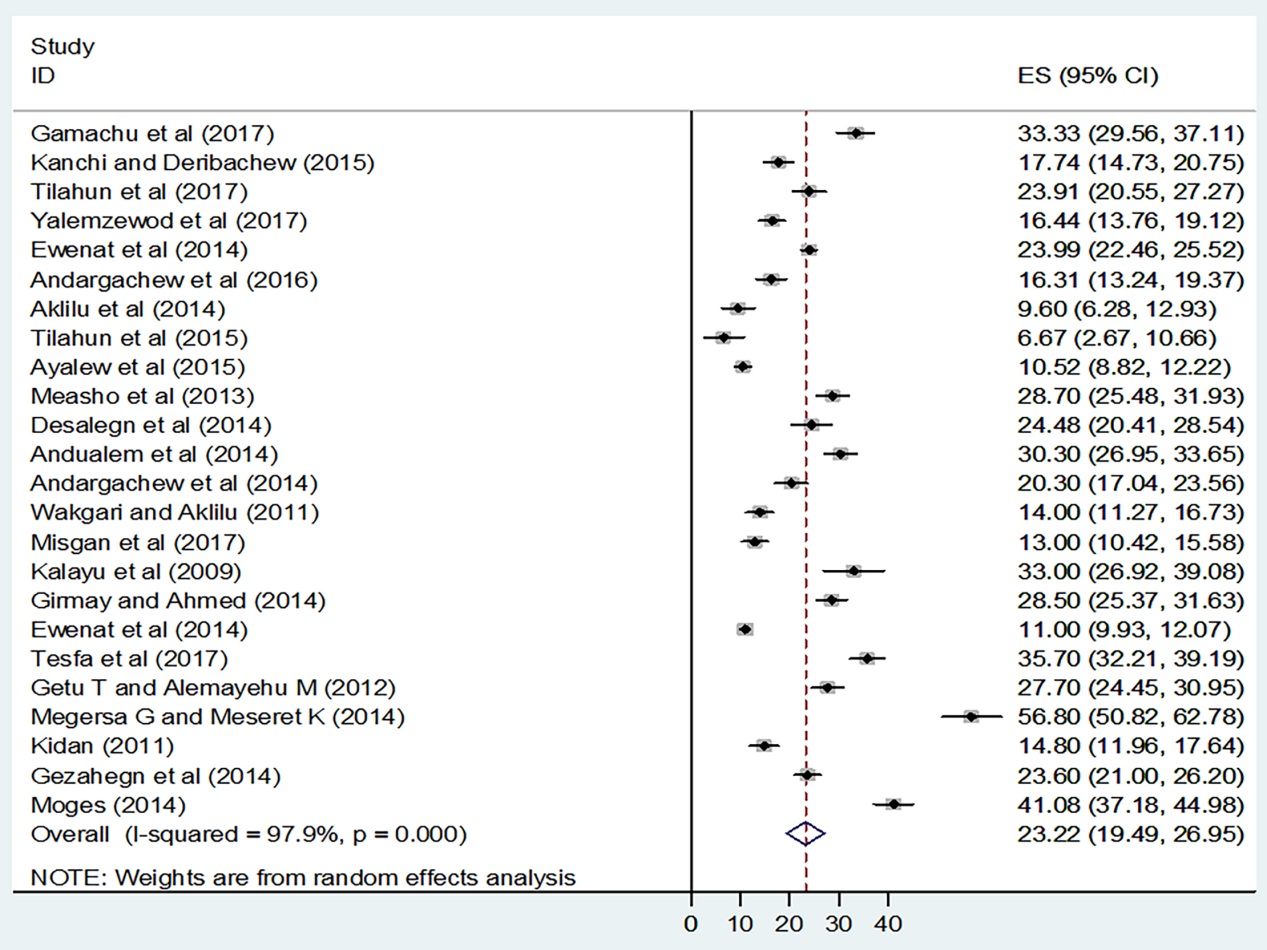
Forest plot of the pooled prevalence of khat chewing in Ethiopian universities, 2017.

As shown in the forest plot, the result of the 24 included studies revealed that the pooled prevalence of khat chewing among university students in Ethiopia was 23.22% (95% CI: 19.5, 27.0%) (**Fig 2**). Nonetheless, extreme heterogeneity was exhibited across the studies and uncovered by I^2^ statistic (I^2^ = 97.9, p value < 0.001). As a result, a random effect model was employed to estimate the pooled prevalence of khat chewing among university students in Ethiopia. In addition, we performed a univariate meta-regression model to identify the possible sources of heterogeneity, by considering different factors associated with the heterogeneity, such as publication year and sample size, but none of these variables was found to be statistically significant (**Table 2**). Beggs’ and Eggers’ tests revealed the presence of statistically significant publication bias (p = 0.008) and (p = 0.001) respectively. Therefore, we were forced to do Trim and Fill analysis to adjust the final pooled estimate.

**Table 2:** Related factors with heterogeneity of khat chewing prevalence among university students in the current meta-analysis (based on a univariate meta-regression model).

Variables	Coefficient	P-value
Publication year	-0.6	0.8
Sample size	0.00054	0.86

### Subgroup analysis

In addition, in this meta-analysis, we performed subgroup analysis based on the regions where the studies were conducted and sample size of the studies. Accordingly, the highest prevalence was observed in Oromia region with a prevalence of 31.6% (95% CI: 21.2, 41.9) followed by SNNP, 24.7% (95% CI: 13.3, 36.1) and others, 21.6% (95% CI: 15.0, 28.3) (**Table 3**). With regard to sample size, the prevalence of khat chewing was slightly higher in studies having a sample size≥ 700, 23.6.2% (95% CI: 18.0, 29.1) as compared to those having a sample size< 700, 23.0% (95% CI: 17.5, 28.6) (**Table 3**).

**Table 3:** Subgroup prevalence of khat chewing in Ethiopian universities, 2017 (n = 24).

Variables	Characteristics	Number of studies	Prevalence with 95%
Region	Oromia	5	31.6(21.2, 41.9)
	Amhara	7	18.1(12.4, 23.8)
	SNNPR	5	24.7(13.3, 36.1)
	Others	7	21.6(15.0, 28.3)
Sample size	< 700	14	23.0(17.5, 28.6)
	≥ 700	10	23.6.2(18.0, 29.1)
Overall		24	23.22 (19.5, 27.0)

#### Predictors of khat chewing practice among university students

**Fig 3:**
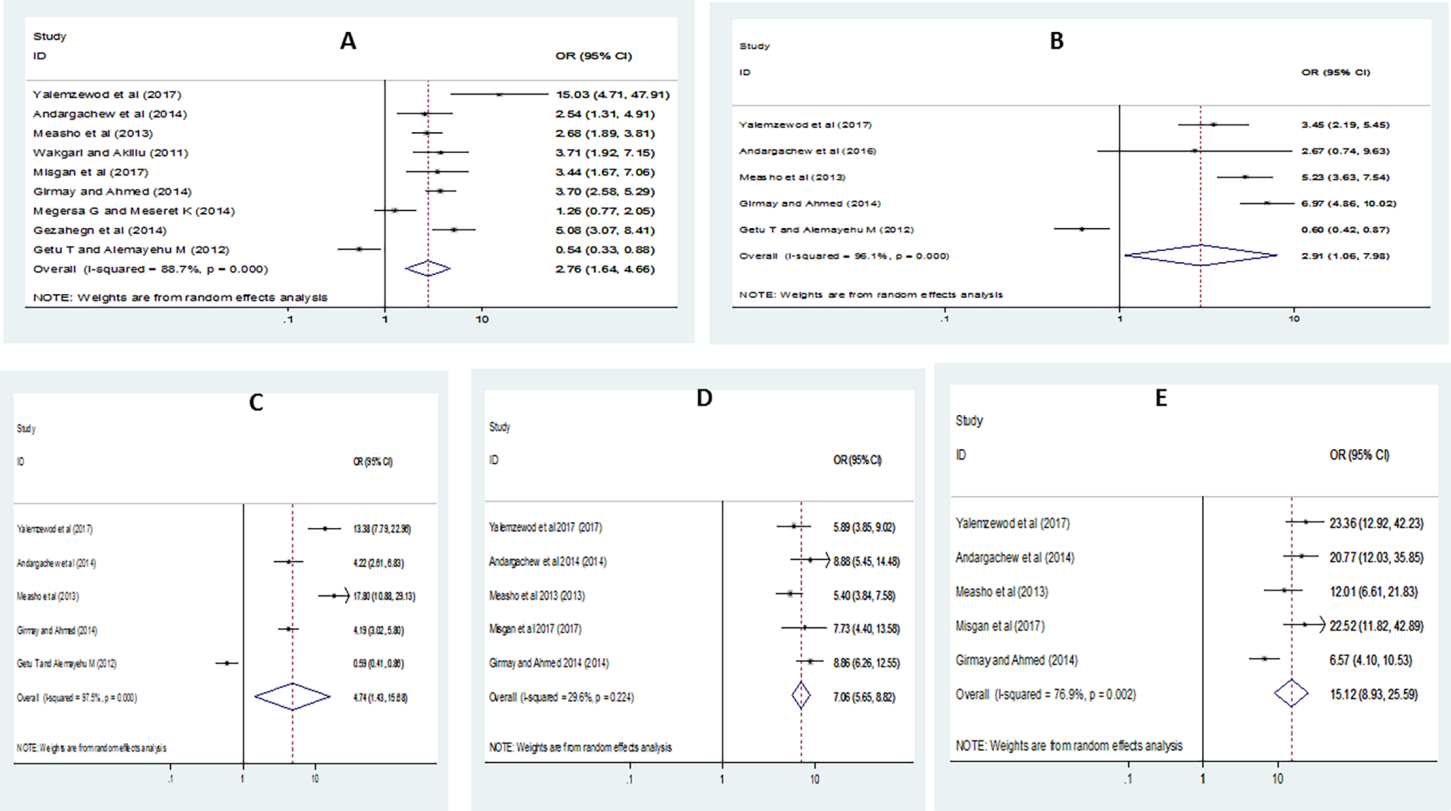
Forest plot depicting pooled odds ratio (log scale) of the associations between khat chewing and its purported determinants (**A**: Gender, **B**: Family khat chewing habit, **C**: Friend khat chewing practice, **D**: Alcohol drinking habit, **E**: Cigarette smoking habit).

Using a total of nine studies [6, 19, 20, 26, 29, 36, 39, 63, 64] with data that can be analyzed, we have examined the associations of gender, family khat chewing practice, friend khat chewing habit, alcohol drinking as well as cigarette smoking habit with khat chewing practice of the university students (**Fig 3**). We also performed sensitivity analysis for each of the factors, but none of studies has shown significant differences in all the analyses.

Hence, from the nine studies, we found that gender was significantly associated with khat chewing practice among the students, odds ratio 2.76 (95% CI 1.64–4.63). In epidemiological terms, this shows us that male students were 2.76 times more likely to chew khat as compared to their female counterparts (**Fig 3A**). Five studies also indicated that the presence of khat-chewing family members and friends was strongly associated with the khat chewing practice of students in the universities (**Fig 3B and 3C**). Those students having family members and friends chewing khat were 2.91 and 4.74 times more likely to chew khat as compared to their counterparts respectively; for family members, odds ratio 2.91 (95% CI 1.06, 7.98) and for friends, odds ratio 4.74 (95% CI 3.48, 13.06).

Moreover, results from the meta-analyses of the studies (**Fig 3D and 3E**) have also revealed that the use of other substances was a significant factor associated with khat chewing practice of the university students. Students with alcohol drinking habit were about seven times more likely to chew khat as compared to those students with no alcohol drinking habit, odds ratio 7.06 (95% CI 5.65, 8.82). Similarly, university students who have been smoking cigarette chewed khat 15.11 times more likely in comparison with those who have not been smoking cigarette odds ratio 15.11 (95% CI 8.96, 25.51).

## Discussion

To the best of our knowledge, this meta-analysis is the first of its kind in Ethiopia to estimate the pooled prevalence and predictors of khat chewing practice among university students. University students commonly practice khat chewing than the general population due to different reasons. Therefore, estimating the prevalence and identifying predictors of khat chewing among these vulnerable groups will be an input to policy makers and program planners to take appropriate interventions.

The overall prevalence of khat chewing practice among university students obtained from this study indicated that almost more than one in five 23.22% (95% CI: 19.5, 27.0%) students have been engaged in the use of this substance. It is evident that khat chewing practices bring about a myriad of psychological, socio-economic, and health consequences affecting a community and a country at large [3–5, 12, 13, 65]. The WHO considered khat as a substance of abuse with no therapeutic potential resulting in psychological dependence pinpointing as if the problem is regional and may best be controlled at that level [66]. Despite the fact that the plant is considered as a controlled/illegal substance in countries like South Africa, China, Germany, Canada, and USA, khat chewing is legal in some nations such as Ethiopia, Somalia, Djibouti, Kenya, Uganda and Yemen. Hence, one can note that it is becoming a burden on poor countries which are already a way behind in development.

Although a similar study (meta-analysis) on this specific research question has not yet been conducted on the area, the prevalence reported in the present study is in trajectory with a systematic review carried out in UK [67], (15–30%). However, the prevalence of khat chewing is lower than that reported for Yemen [68] (80% for males, and 50% for females), the possible justification for this variation could be due to the difference in study area and setting with variations in socio-cultural values and norms as well as religious beliefs as it is supported by different studies [19, 21, 26]. Another possible explanation could be attributed to the difference in socio-economic status and research methodology. Likewise, our finding is much lower than studies conducted at Eldoret Kenya (68.9%) [69] and Nigeria [70]. This variation may be due to the differences in culture, study resign and policies of the respective universities, availability of substances, surrounding community, culture, and how well the study participants understood the health risks of khat chewing.

The subgroup analysis of this study indicated that the prevalence of khat chewing practice among university students significantly varied across regions of the country. The highest prevalence of khat chewing was observed in Oromia region, 31.6% (95%CI: 21.2, 41.9%) followed by SNNPR region, 24.7% (95% CI: 13.3, 36.1%) whereas the lowest prevalence was observed Amhara region with a prevalence of 18.1% (95%CI: 12.4, 23.8%).The possible justifications for this variation could be due to social, religious, and cultural differences across the regions. The other possible justification for the higher prevalence of khat chewing practice in Oromia region could be due to the differences in the study settings, such as access to khat, and factors outside the university environment, for example, in Jimma town chewing khat is more common and is normalized by the community [71]. With regard to sample size, the prevalence of khat chewing was slightly higher in those studies having a sample size ≥ 700. This shows us that sample size variation has a great effect on our estimation.

The present study was also aimed to identify the predictors of khat chewing practice among Ethiopian university students based on the reports of primary studies. In the current meta-analysis, being male, presence of khat chewing family member, friend khat chewing habit, alcohol drinking, and cigarette smoking habit were found to be predictors of khat chewing practice. Accordingly, in this study khat-chewing habit was significantly higher in males as compared to female students. This finding is consistent with studies conducted in Saudi Arabia [72], and Eastern Ethiopia [57]. This significant difference between male and female students could be explained by the fact that in Ethiopia females are less exposed to khat chewing practice than males due to the common trend of social and cultural restrictions to females towards khat chewing practice[35].

The presence of khat-chewing family members and friends were also another strong predictor of khat chewing practices among universities. Students who had family members and friends engaged in the khat chewing habit were predisposed to the practice of khat chewing. This finding is in line with different studies carried out on substance abuse [56, 72–74]. The possible reason for the association may be because of social influence and peer pressure that the adult segment of a population has a natural tendency to exercise and reproduce what they have seen in their family and friends. Another possible explanation could be university students usually started kaht chewing practice during adolescent period; they begin it due to friends, and peers influence[75, 76]. Furthermore, during young age, individuals have a higher tendency to imitate and exercise what they observe their elders deed.

Moreover, in this meta-analysis we observed that those students who had ever drunk alcohol and ever smoked cigarette were more likely to practice khat chewing as compared to their counterparts. This finding is supported by previous studies conducted in Kenya and Ethiopia[74, 77]. These studies reported that history of alcohol consumptions and cigarette smoking were positively associated with khat chewing practice. The possible explanation could be because of individuals’ need of khat for stimulant effect during alcohol consumption and cigarette smoking.

### Limitations of the study

The first limitation of this study was only English articles or reports were considered to conduct this international based review. In addition, all of the studies included in this review were cross-sectional in nature as a result; the outcome variable might be affected by other confounding variables. The majority of the studies included in this review had a small sample size. Therefore, this factor could affect the estimated report. Furthermore, this meta-analysis represented only studies reported from seven regions of the country. Therefore, the regions may be under-represented due to the limited number of studies included.

### Conclusions

The study found that the prevalence of khat chewing among university students was quite common, with slightly more than 1 in 5 students engaging in the use of this substance. Being male, family khat chewing practice, friend’s khat chewing habit, alcohol drinking, and cigarette smoking were found to be predictors of khat chewing. Therefore, based on our findings, we strongly recommend that a special consideration shall be given to university students. Moreover, the university administrators in collaboration with other government and non-government organization stakeholders’ should launch health education programs about the health hazards of khat to the university students.

## Supporting information

S1 Table PRISMA checklist. (DOC)

S2 Table The predictors of khat chewing data abstraction format. (DOCX)

## Abbreviations

PRISMA: Preferred Reporting Items of Systematic Reviews and Meta-Analysis
WHO: World Health Organization
(SNNPR): Southern Nations, Nationalities and peoples of Region
USA: United States of America
