# Supplementary material for: Prevalence and predictors of khat chewing among Ethiopian university students: A systematic review and meta-analysis
Source: PLoS One. 2018 Apr 12;13(4):e0195718. doi: 10.1371/journal.pone.0195718 (PMC5896981; doi:10.1371/journal.pone.0195718)
Supplement: S2 Table — (DOCX) [file pone.0195718.s002.docx]

| Author | Publication year | A | B | C | D |
| --- | --- | --- | --- | --- | --- |
| Yalemzewod et al | 2017 |  |  |  |  |
| Andargachew et al | 2014 |  |  |  |  |
| Measho et al | 2013 |  |  |  |  |
| Wakgari and Aklilu | 2011 |  |  |  |  |
| Misgan et al | 2017 |  |  |  |  |
| Girmay and Ahmed | 2014 |  |  |  |  |
| Megersa G and Meseret K | 2014 |  |  |  |  |
| Gezahegn et al | 2014 |  |  |  |  |
| Getu T and Alemayehu M | 2012 |  |  |  |  |

**Factor 1.** Data extraction format for Gender

**Key**

| gender | Khat chewing | |
| --- | --- | --- |
|  | Yes | No |
| Male | A | B |
| Female | C | D |

Female is reference

**Factor 2.** Data extraction format for family khat chewing practice

| a Author | Publication year | A | B | C | D |
| --- | --- | --- | --- | --- | --- |
| Yalemzewod et al | 2017 |  |  |  |  |
| Andargachew et al | 2016 |  |  |  |  |
| Measho et al | 2013 |  |  |  |  |
| Girmay and Ahmed | 2014 |  |  |  |  |
| Getu T and Alemayehu M | 2012 |  |  |  |  |

**Key**

| Family khat chewer |  | Khat chewing of students | |
| --- | --- | --- | --- |
|  |  | Yes | No |
|  | Yes | A | B |
|  | No | C | D |

**No is reference**

**Factor 3.** Data extraction format for friend khat chewing practice

| Author | Publication year | A | B | C | D |
| --- | --- | --- | --- | --- | --- |
| Yalemzewod et al | 2017 |  |  |  |  |
| Andargachew et al | 2014 |  |  |  |  |
| Measho et al | 2013 |  |  |  |  |
| Girmay and Ahmed | 2014 |  |  |  |  |
| Getu T and Alemayehu M | 2012 |  |  |  |  |

**Key**

| Friend khat chewer |  | Khat chewing of students | |
| --- | --- | --- | --- |
|  |  | Yes | No |
|  | Yes | A | B |
|  | No | C | D |

No is reference

**Factor 4.** Data extraction format for alcohol consumption

| Author | Publication year | A | B | C | D |
| --- | --- | --- | --- | --- | --- |
| Yalemzewod et al 2017 | 2017 |  |  |  |  |
| Andargachew et al 2014 | 2014 |  |  |  |  |
| Measho et al 2013 | 2013 |  |  |  |  |
| Misgan et al 2017 | 2017 |  |  |  |  |
| Girmay and Ahmed 2014 | 2014 |  |  |  |  |

**Key**

| Alcohol consumption |  | Khat chewing of students | |
| --- | --- | --- | --- |
|  |  | Yes | No |
|  | Yes | A | B |
|  | No | C | D |

No is reference

**Factor 5.** Data extraction format for cigarette smoking

| Author | Publication year | A | B | C | D |
| --- | --- | --- | --- | --- | --- |
| Yalemzewod et al | 2017 | 50 | 18 | 71 | 597 |
| Andargachew et al | 2014 | 63 | 24 | 56 | 443 |
| Measho et al | 2013 | 55 | 15 | 160 | 524 |
| Misgan et al | 2017 | 32 | 20 | 40 | 563 |
| Girmay and Ahmed | 2014 | 60 | 30 | 168 | 552 |

**Key**

| Alcohol consumption |  | Khat chewing of students | |
| --- | --- | --- | --- |
|  |  | Yes | No |
|  | Yes | A | B |
|  | No | C | D |

No is reference

The data have been abstracted by Microsoft Excel and exported (pasted to the data editor of STATA 14) then meta-analyzed using the command “metan a b c d,or xlab(1,5) label(namevar=aurhor,yearvar=publication yea) random texts(110) nowt” to produce the forest plot for the five factors
